# The Southern African Society for Virology

**DOI:** 10.4102/sajid.v39i1.672

**Published:** 2024-08-30

**Authors:** Wolfgang Preiser, Marietjie Venter, Nicola A. Page, Felicity J. Burt

**Affiliations:** 1 Division of Medical Virology, Faculty of Medicine and Health Sciences, Stellenbosch University, Cape Town, South Africa; 2 National Health Laboratory Service, Cape Town, South Africa; 3 Emerging Viral Threats, One Health Surveillance and Vaccines (EViTOH) Division, Infectious Disease and Oncology Research Institute (IDORI), University of the Witwatersrand, Johannesburg, South Africa; 4 Centre for Enteric Diseases, National Institute for Communicable Diseases, Division of the National Health Laboratory Service, Johannesburg, South Africa; 5 Pathogen Research Laboratory, Division of Virology, Faculty of Health Sciences, University of the Free State, Bloemfontein, South Africa; 6 National Health Laboratory Service, Bloemfontein, South Africa

The newly founded Southern African Society for Virology (SASV) (logo shown in Figure 1) was accepted as a new member of the Federation of Infectious Diseases Societies of Southern Africa (FIDSSA)^1^ at its recent congress.^2^

**FIGURE 1:**
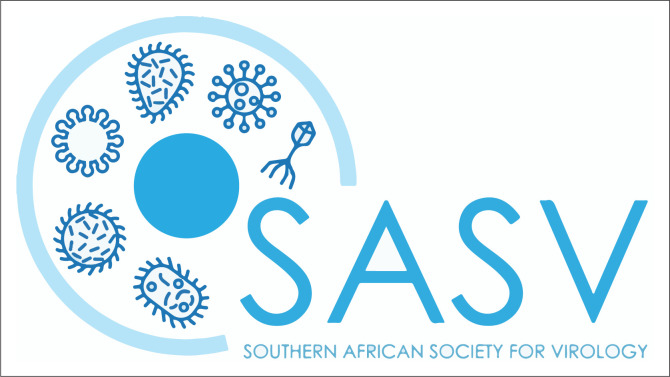
Southern African Society for Virology logo^4^.

## Purpose

Southern African Society for Virology is southern Africa’s first and only virology society. The SASV aims to promote research, education and excellence in virology throughout southern Africa, encompassing all scientists and practitioners working in virology in and beyond the medical field. Southern African Society for Virology welcomes members from various professions and disciplines, including medical (pathologists and medical scientists in public and private sectors), veterinary, plant, environmental and basic virology, plus related sciences and practices.

Southern African Society for Virology seeks to attract members from across southern Africa, including South Africa, Lesotho, Swaziland, Namibia, Botswana, Zimbabwe, Mozambique as well as Angola, Malawi, Zambia and Tanzania.

## Aims

The SASV aims to:

- advance knowledge and information exchange in applied and basic virology
- organise conferences, workshops and seminars to disseminate scientific knowledge and support professional development
- advocate for public policies and funding to support virological research and training
- develop, publish and distribute scientific and educational materials
- build relationships with international virology organisations to enhance global scientific exchange and collaboration
- foster collaboration among virologists and those working in related fields
- improve the quality and standardisation of methods and support scientific and technological development
- contribute to surveillance activities (e.g. antiviral drug resistance, vaccine efficacy, pathogen evolution)
- develop guidance documents and recommendations for preventing, managing and treating viral diseases in humans, animals and plants
- provide advice and input on virological issues to professional organisations, government departments and other bodies, assisting in policy and guideline formulation.

## Membership and committees

Membership categories are aligned with FIDSSA. Postgraduate students of relevant fields enjoy free membership. An Executive Committee manages SASV’s affairs, while a Steering Committee offers advice and provides strategic direction. It will include members representing different virology fields, professional groups (such as medical doctors, veterinarians, and scientists), and reflecting the diversity of Southern Africa’s population, regions, and institutions. Sub-Committees will be formed as needed for specific tasks.

## Congresses

Southern African Society for Virology, working alongside other FIDSSA societies, will co-organise the medical virology tracks, sessions, and workshops of the biennial FIDSSA Congresses. This will make participation in these events more attractive for medical virologists.

Between biennial FIDSSA Congresses, SASV will organise Virology Africa Congresses. Primarily scientific in nature, these congresses cover all fields of virology, focus on student participation, and have a proud tradition of providing African virologists with the opportunity to network and interact with internationally recognised presenters. Building on the famed Berg-en-Dal meetings organised by Prof. Barry Schoub (National Institute for Communicable Diseases), Profs Anna-Lise Williamson and Ed Rybicki (University of Cape Town) organised the congresses in 2005, 2011, 2015 and 2020 and were joined by Prof. Marietjie Venter (University of the Witwatersrand) for Virology Africa 2024. This attracted 280 attendees including more than 100 students and emerging researchers from around Africa, sponsored by the Poliomyelitis Research Foundation (PRF) and the Bill and Melinda Gates Foundation (BMGF).

While highly successful and formative for virology in southern Africa, the Virology Africa Congresses have not previously been backed by a Society. Following deliberations with Duncan Steele (BMGF) and an invitation from FIDSSA to establish a virology society, an online survey of more than 70 senior virologists established strong interest for this. In 2023, a Virology Group was established in FIDSSA coordinated by Marietjie Venter and Wolfgang Preiser. When presented at Virology Africa 2024, the idea of SASV was met with enthusiasm by virologists across all fields.

The virology scientific committee of FIDSSA 2024 wrote the SASV constitution, became SASV’s founding members and are serving as its interim executive committee until the first Annual General Meeting (AGM)^3^: Marietjie Venter as Chairperson, Felicity Burt as Deputy Chairperson, Nicola Page as Treasurer and Wolfgang Preiser as Secretary.

By becoming their organiser, SASV will provide a firm foundation for the future of Virology Africa Congresses. Southern African Society for Virology already has established links with the American Society of Virology and World Society of Virology.

## Inviting Virologists to become involved

We warmly invite you to join SASV (easily, via the FIDSSA website: sign up and choose SASV as your society)! Whether you are a medical or veterinary virologist in public or private practice, a plant virologist, or a scientist working in applied or basic virological sciences; and whether you are an accomplished expert or specialist in your field or studying to become one, you are welcome and can play a role in shaping the future of virology in our region!

Furthermore, we urge you to become actively involved: participate in SASV meetings, volunteer on SASV Committees and Sub Committees and serve on Conference Organising Committees. We are currently seeking nominations for Steering Committee membership from all fields of Virology. Please also share your ideas and suggestions about topics SASV should consider, about workshops and other activities, including those focused on students.

Southern African Society for Virology is yours and your input is welcome to help in ensuring a bright future for virology in all its facets in the southern African region!
